# Acute administration of catalase targeted to ICAM-1 attenuates neuropathology in experimental traumatic brain injury

**DOI:** 10.1038/s41598-017-03309-4

**Published:** 2017-06-19

**Authors:** Evan M. Lutton, Roshanak Razmpour, Allison M. Andrews, Lee Anne Cannella, Young-Jin Son, Vladimir V. Shuvaev, Vladimir R. Muzykantov, Servio H. Ramirez

**Affiliations:** 1 Department of Pathology and Laboratory Medicine, The Lewis Katz School of Medicine at Temple University, Philadelphia, PA 19140 USA; 2 Shriners Hospitals Pediatric Research Center, The Lewis Katz School of Medicine at Temple University, Philadelphia, PA 19140 USA; 3 Center for Substance Abuse Research, The Lewis Katz School of Medicine at Temple University, Philadelphia, PA 19140 USA; 4 Department of Systems Pharmacology and Translational Therapeutics, Perelman School of Medicine, University of Pennsylvania, Philadelphia, PA 19104 USA

## Abstract

Traumatic brain injury (TBI) contributes to one third of injury related deaths in the US. Treatment strategies for TBI are supportive, and the pathophysiology is not fully understood. Secondary mechanisms of injury in TBI, such as oxidative stress and inflammation, are points at which intervention may reduce neuropathology. Evidence suggests that reactive oxygen species (ROS) propagate blood-brain barrier (BBB) hyperpermeability and inflammation following TBI. We hypothesized that targeted detoxification of ROS may improve the pathological outcomes of TBI. Following TBI, endothelial activation results in a time dependent increase in vascular expression of ICAM-1. We conjugated catalase to anti-ICAM-1 antibodies and administered the conjugate to 8 wk old C57BL/6J mice 30 min after moderate controlled cortical impact injury. Results indicate that catalase targeted to ICAM-1 reduces markers of oxidative stress, preserves BBB permeability, and attenuates neuropathological indices more effectively than non-targeted catalase and anti-ICAM-1 antibody alone. Furthermore, the study of microglia by two-photon microscopy revealed that anti-ICAM-1/catalase prevents the transition of microglia to an activated phenotype. These findings demonstrate the use of a targeted antioxidant enzyme to interfere with oxidative stress mechanisms in TBI and provide a proof-of-concept approach to improve acute TBI management that may also be applicable to other neuroinflammatory conditions.

## Introduction

Traumatic brain injury (TBI) is a prevalent healthcare concern with an estimated 1.7 million cases occurring annually in the US alone^1, 2^. In addition to civilian sports-related injuries, motor vehicles accidents, and falls, recent warfare has increased the number of Veterans experiencing TBI, further demonstrating the need for effective therapeutics that can be administered acutely following injury in the field^3, 4^. Several preclinical and clinical studies have been conducted to assess the benefit of monotherapies and combination therapies in TBI; however, few have demonstrated success in improving patient outcomes^5, 6^. Consequently, TBI patients are limited to supportive treatment options and rehabilitation, with extensive recovery times and often-permanent disability.

The pathophysiology of TBI has been characterized with two broad phases^7^. Primary injury occurs at the moment of impact. Primary injury can involve contusion, diffuse axonal injury, brain swelling and intracranial hemorrhage, which invariably results in focal necrotic cell death^8^. Secondary injury, which includes blood-brain barrier (BBB) disruption, neuroinflammation, oxidative damage, and glutamate excitotoxicity, is not well controlled and can lead to exacerbated injury, progressive neurodegeneration, and delayed cell death^9^. These processes begin at the time of the traumatic event and continue to contribute to cerebral damage for days and weeks following injury^10^. Remarkably, persistently activated microglia, an indication of chronic neuroinflammation, have been identified in parasagittal and hippocampal white matter in long-term survivors of head injury up to 16 years after a TBI was sustained^11^. Furthermore, chronic inflammation after TBI can predispose individuals to comorbidities including substance use disorder, depression, and post-traumatic stress disorder^12–15^. The dynamic pathophysiology and extensive morbidity of TBI, in addition to limited current treatment modalities, demonstrate the need for therapeutic interventions targeted against specific secondary injury processes.

Oxidative stress reactions occur early following TBI, within minutes of mechanical impact, and contribute to propagating injury mechanisms including inflammation, excitotoxicity, and cell death^16–19^. Innate mechanisms including the endogenous expression of antioxidant enzymes, catalase and superoxide dismutase, and the antioxidant glutathione balance and control oxidative stress; however, the extensive and rapid production of free radicals and reactive oxygen species (ROS) that occurs in brain injury can readily overwhelm the system^20^. Acute intervention of oxidative stress processes could limit the negative effects of secondary injury mechanisms on TBI outcome. A challenge to TBI treatment, and the treatment of any central nervous system (CNS) disorder, is drug delivery to and across the BBB^21^. Notably, the endothelial cell layer that constitutes the luminal most component of the BBB represents an important therapeutic target in conditions involving oxidative stress and inflammation, such as TBI^22, 23^. Excessive production of ROS can cause endothelial dysfunction and activation, which is manifested by increased BBB permeability and upregulation of cellular adhesion molecules (e.g. ICAM-1, VCAM-1)^24, 25^. Targeting therapeutics to endothelial surface determinants including these molecules may help maintain BBB integrity and prevent the disruption of the internal CNS milieu, thereby ameliorating inflammatory mechanisms of injury and the subsequent neuropathology that characterizes cerebral damage in TBI^13, 26^. Endothelial targeting of biotherapeutics using affinity ligands, such as antibodies to endothelial cell adhesion molecules, has been studied in other settings including for experimental treatment of acute lung injury; however, endothelial targeting of antioxidant enzymes has not been reported for TBI^6, 27, 28^.

Following TBI, administration of the antioxidant enzyme catalase conjugated to monoclonal antibodies against Intercellular Adhesion Molecule 1 (ICAM-1) provides targeted delivery of catalase to the cerebrovascular endothelium where ICAM-1 is known to be upregulated in response to injury^29, 30^. In the present study, we investigated whether delivery of catalase to ICAM-1 in the context of TBI can interfere with the oxidative stress response to injury to thus preserve BBB integrity and alleviate the neuropathological findings associated with TBI. To our knowledge, this is the first comprehensive assessment of a targeted enzymatic approach to preventing oxidative secondary injury in TBI.

## Results

### Traumatic brain injury causes endothelial and glial activation with increased oxidative damage in the cerebral cortex

**Figure 1:**
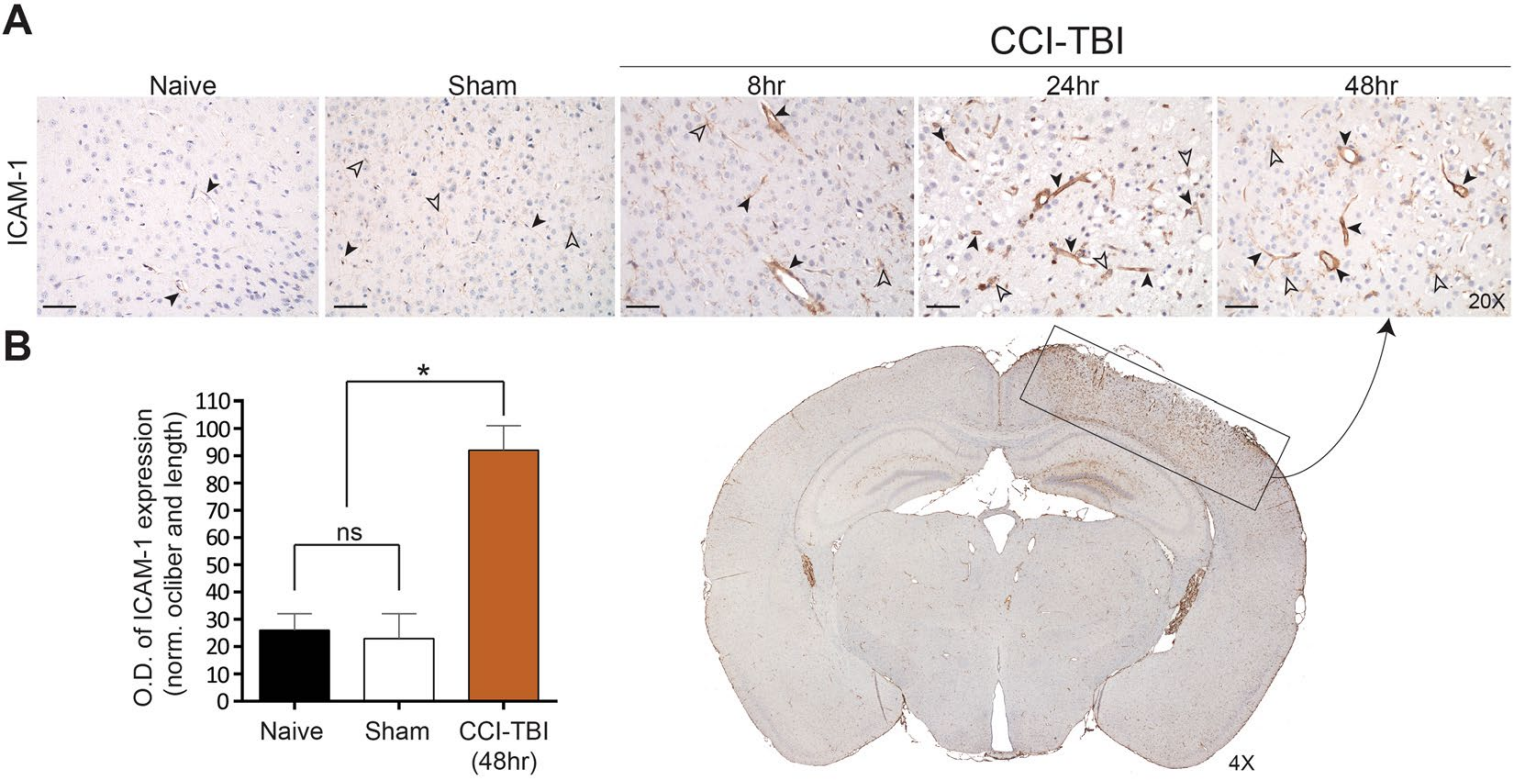
CCI-TBI induces local and temporal increase in ICAM-1 expression in mouse cerebral cortex and underlying brain structures. (**A**) Immunohistochemical detection of ICAM-1 time course of expression in cerebral vasculature (solid arrows) and aberrant expression in astrocytes (open arrows) in area of impact in naive (no craniectomy control), sham (surgical control without impact) and at 8, 24, and 48 hr post-CCI-TBI (20X). Included is a stitched coronal section through the CCI-TBI impact site 48 hrs following CCI-TBI (boxed, 4X). Increased ICAM-1 expression detection can also be visualized in hippocampal regions underlying the impact site. (**B**) Quantification of ICAM-1 staining optical density (OD) normalized to vessel caliber and length for naive, sham, and 48 hr post-CCI-TBI groups. Scale bar equals 50 microns. Data are presented as mean ± SD. (Ordinary one-way ANOVA with multiple comparisons F = 96.35, P < 0.0001).

To model the cerebral damage and vascular disruption that occurs during the primary phase of TBI, the controlled cortical impact model was utilized to deliver a TBI of moderate severity (CCI-TBI) to the cortex of 8 wk old C57BL/6 J mice. This approach has been reported to reproduce histopathological features that reflect what is observed clinically in TBI, consistently between animal trials and in a time-dependent manner^31–33^. The features of moderate severity CCI-TBI that are congruent with the human condition include neurodegeneration, neuroinflammation, and reactive astrogliosis. Furthermore, CCI-TBI in mice mimics cortical tissue loss, acute subdural hematoma, diffuse axonal injury, concussive syndromes, and BBB dysfunction that characterize TBI in humans^34^. Figure 1 demonstrates the temporal upregulation of endothelial surface and activation marker Intercellular Adhesion Molecule 1 (ICAM-1) on endothelial cells of the cortical vasculature in coronal cross sections of the area of impact. Basal level expression can be seen in the naive condition, which is maintained in the surgical sham. ICAM-1 expression detection was greatly increased at 8, 24, and 48 hrs following CCI-TBI (solid arrows). Analysis of optical density of ICAM-1 detection showed a nearly 3-fold increase in ICAM-1 levels at 48 hrs following CCI-TBI when normalized to vascular length and caliber compared to naive and sham controls, respectively (Fig. 1B). ICAM-1 upregulation was also observed in the brain parenchyma, likely by reactive astrocytes (open arrows), supporting the aberrant expression of ICAM-1 in glial cells in the context of CNS pathology as has previously been described^35^. Of note, increased ICAM-1 expression was detected by immunostaining isolated microvessels from the cerebral cortex of mice as early as 4 hrs following CCI-TBI (data not shown). The abrupt and sustained upregulation of ICAM-1 in the cerebral vasculature and within the brain parenchyma at the site of injury identify this molecule as a candidate for targeted intravenous delivery of therapeutic cargo to the brain.

**Figure 2:**
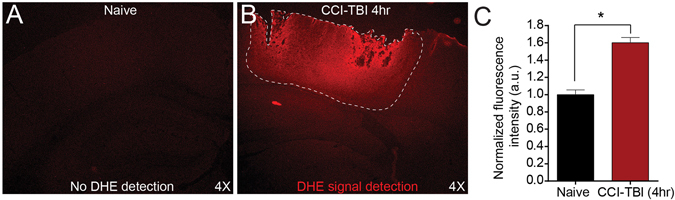
CCI-TBI induces ROS free radical production in ipsilateral cortex area of injury. *In vivo* dihydroethidium staining detection in naive (**A**) vs. 4 hrs following CCI-TBI (**B**) cerebral cortex in area of CCI-TBI impact site (4X). Red fluorescence indicates oxidation of dihydroethidium and *in situ* ROS free radical detection. The impact site and affected ipsilateral cortical tissue is outlined with a white dotted line. ROS detection extends through and beyond the depth of the cortex. (**C**) Quantification of dihydroethidium fluorescence intensity in naive compared to CCI-TBI groups at 4 hrs post-injury. Data are presented as mean ± SD. (Two-tailed unpaired t test with equal SD P = 0.0002, t = 12.83, df = 4).

Furthermore, the generation of free radicals and reactive oxygen species begins nearly instantaneously following injury^16^. By utilizing a novel *in vivo* staining technique, freely permeable dihydroethidium (DHE) was administered by IP injection to observe the presence of endogenous oxidizing agents *in situ* in the injured cortex as early as 4 hrs following CCI-TBI compared to naive controls (Fig. 2). Moderate CCI-TBI gave significantly increased free radical detection compared to naive at 4 hrs after injury as indicated by increased DHE fluorescence intensity in cortical tissue surrounding the area of impact (Fig. 2C). Taken together, these results support the notion that ICAM-1 can be utilized as a target for delivery of antioxidant enzyme therapy directed to areas of injury in the brain.

### Acute administration of anti-ICAM-1/catalase reduces hydrogen peroxide and 3-nitrotyrosine levels in the area of injury

**Figure 3:**
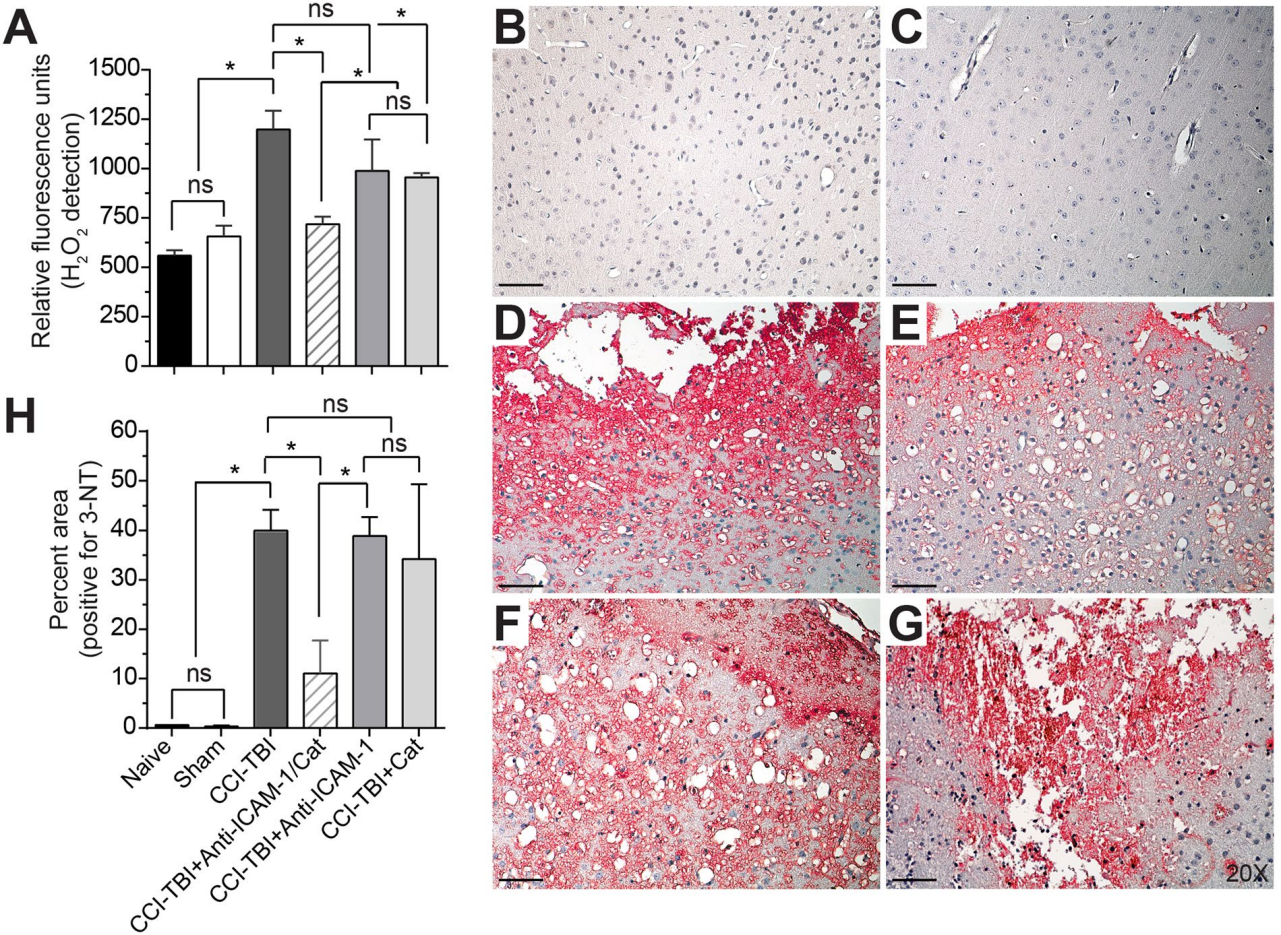
CCI-TBI results in increased hydrogen peroxide production and 3-nitrotyrosine detection in the ipsilateral cortex area of impact. (**A**) OxiSelect™ Hydrogen Peroxide/Peroxidase Assay Kit was used to measure hydrogen peroxide levels in fresh mouse cortical tissue ipsilateral to the area of impact 4 hrs following CCI-TBI, CCI-TBI+anti-ICAM-1/catalase, catalase only, and antibody only administration or from naive and sham controls. The naive condition provides basal hydrogen peroxide levels in uninjured cortical tissue which are not significantly different from sham. CCI-TBI results in a nearly 3-fold increase in hydrogen peroxide level detection. Anti-ICAM-1/catalase quenches hydrogen peroxide levels in the area of injury to physiological levels comparable to that of naive and sham, an effect not achieved with anti-ICAM-1 antibody and catalase alone. Immunohistochemical chomogen staining of 3-NT demonstrates increased tyrosine nitration following CCI-TBI (**D**) compared to naive and sham controls (**B** and **C**, respectively). Anti-ICAM-1/catalase significantly decreased 3-NT detection (**E**). 3-NT levels in anti-ICAM-1 antibody and catalase only groups did not significantly differ from CCI-TBI alone (**F** and **G**, respectively). Positive signal detection was pseudo-colored red for better visualization. All images taken at 20X. Scale bar equals 50 microns. (**H**) Quantification of 3-NT signal detection was analyzed by percent area and normalized to total tissue area per image. Data are presented as mean ± SD. (Ordinary one-way ANOVA with multiple comparisons for hydrogen peroxide data F = 25.95, P < 0.0001 and 3-NT data F = 21.07, P < 0.0001).

To test the hypothesis that targeted delivery of antioxidant enzyme therapy could reduce levels of ROS and subsequent secondary injury mechanisms following TBI, conjugates of anti-ICAM-1 antibodies covalently linked to recombinant catalase were administered to mice 30 min following moderate CCI-TBI. Catalase is an antioxidant enzyme that converts hydrogen peroxide to oxygen and water. To assess the efficacy of anti-ICAM-1/catalase in reducing hydrogen peroxide in TBI, levels were measured at 4 hrs following injury for all experimental groups. CCI-TBI significantly increased hydrogen peroxide in the cortex ipsilateral to the injury site compared to the naive and sham conditions, and anti-ICAM-1/catalase administration significantly reduced hydrogen peroxide compared to injury alone to levels not significantly different from baseline (Fig. 3A). Anti-ICAM-1 antibody administered alone following CCI-TBI did not reduce hydrogen peroxide production following injury. Interestingly, catalase administered alone did significantly reduce hydrogen peroxide compared to untreated CCI-TBI; however, levels were still significantly elevated compared to naive and sham groups. Based on these results, acute intervention with anti-ICAM-1/catalase demonstrates feasibility to reduce hydrogen peroxide levels to in the brain within physiological parameters in CCI-TBI. Furthermore, anti-ICAM-1/catalase effectively reduces the extent of tyrosine nitration following CCI-TBI (Fig. 3B–H). Tyrosine nitration occurs as a result of peroxynitrite production and is a well-defined marker of oxidative stress. Levels of 3-nitrotyrosine (3-NT) were measured by immunohistological staining and demonstrate significant increases in 3-NT levels following CCI-TBI with positive staining normalized to total tissue area. Nitration of tyrosine residues was limited with anti-ICAM-1/catalase administration to an extent that was not achieved by catalase or anti-ICAM-1 antibody given alone (Fig. 3E–G). The reduction of 3-nitrotyrosine levels with administration of anti-ICAM-1/catalase suggests that treatment conveys protection against oxidative damage in this experimental model of TBI.

### Anti-ICAM-1/catalase preserves BBB integrity following CCI-TBI

The neuropathology associated with TBI is a result of cell death and the activation of glia, including microglia and astrocytes^9^. Subsequently, the release of inflammatory mediators and secondary messengers in the brain results in the recruitment of peripheral immune cells, chronic inflammatory activation, and continued neuronal damage. Additionally, cellular and biochemical insults such as necrosis and oxidative stress disrupt the neuronal microenvironment and support the propagation of injury^20^.

**Figure 4:**
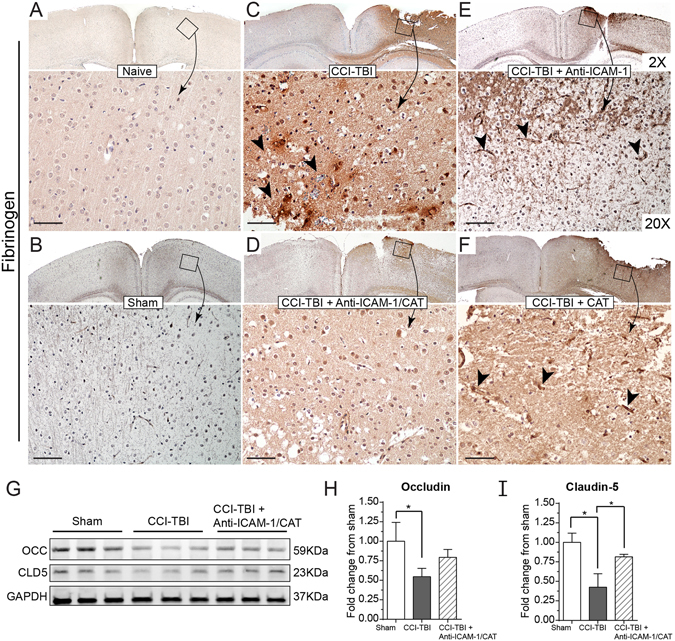
CCI-TBI increases BBB permeability to fibrinogen and decreases tight junction protein detection with a rescue or preservation of barrier function with anti-ICAM-1/catalase. Immunohistochemical detection of plasma protein fibrinogen in brain parenchyma at 48 hr following moderate CCI-TBI. (**A,B**) Naive and sham controls show absent fibrinogen detection in the brain parenchyma, as the BBB is healthy and intact. Absence of staining maintained at 20X. (**C**) Following CCI-TBI, BBB hyperpermeability permits the extravasation of fibrinogen into the brain tissue. Black arrowheads indicate areas of dense fibrinogen staining in the perivascular space. (**D**) Anti-ICAM-1/catalase reduces parenchymal and perivascular fibrinogen detection in the brain by 48 hrs post-CCI-TBI. (**E**,**F**) Anti-ICAM-1 antibody and catalase alone do not appear to reduce fibrinogen extravasation as indicated by intense fibrinogen detection in the impact site with dense staining in perivascular space. Scale bar equals 50 microns. (Top panels 2X, bottom panels 20X). (**G**) Western blot analysis of tight junction protein expression for occludin and claudin-5 in the cortex ipsilateral to the site of CCI-TBI for sham, CCI-TBI and CCI-TBI+anti-ICAM-1/catalase groups. The whole membrane was cut at 75 kDa and between 37 and 25 kDa to minimize antibody use. Membranes were separately probed for occludin and claudin-5. The membrane probed for occludin was striped, reblocked, and then probed for GAPDH. Full length blots are presented in Supplementary Figure 1. (**H**,**I**) Densitometry quantification for occludin and claudin-5 normalized to GAPDH presented as mean ± SD (Ordinary one-way ANOVA with multiple comparisons occludin F = 5.779, P = 0.0399. Claudin-5 F = 17.42, P = 0.0032).

The effect of anti-ICAM-1/catalase conjugate therapy on the BBB was first explored with the hypothesis that combating oxidative stress acutely following CCI-TBI would maintain or rescue barrier function and prevent subsequent neuropathology. BBB integrity was assessed by two permeability assays. First, brain tissue was collected at 48 hrs following CCI-TBI and prepared for immunohistochemical staining of the plasma protein fibrinogen (MW 340 kDa)^36^. The naive and sham conditions gave baseline (absent) staining of fibrinogen (Fig. 4A,B), which was greatly increased in the brain parenchyma and perivascular space following CCI-TBI reflecting loss of BBB integrity (Fig. 4C). Additionally, with CCI-TBI, fibrinogen was observed to accumulate in perivascular space (black arrows). Acute administration of anti-ICAM-1/catalase rescued BBB integrity toward what is seen in the naive, with less fibrinogen detection in the tissue and absent perivascular accumulations (Fig. 4D). This effect was not achieved when anti-ICAM-1 or catalase were administered alone highlighting the importance of targeted delivery of catalase (Fig. 4E,F). Western blot analysis of tight junction protein (TJP) expression in the cortex ipsilateral to the area of injury further demonstrates the consequences of CCI-TBI on the BBB with significant decreases in the detection of TJPs occludin and claudin-5 at 48 hrs following CCI-TBI. Administration of anti-ICAM-1/catalase restored TJP levels for claudin-5 and trended toward sham expression levels for occludin (Fig. 4G–I). Full length blots are presented in Supplemental Figure 1.

**Figure 5:**
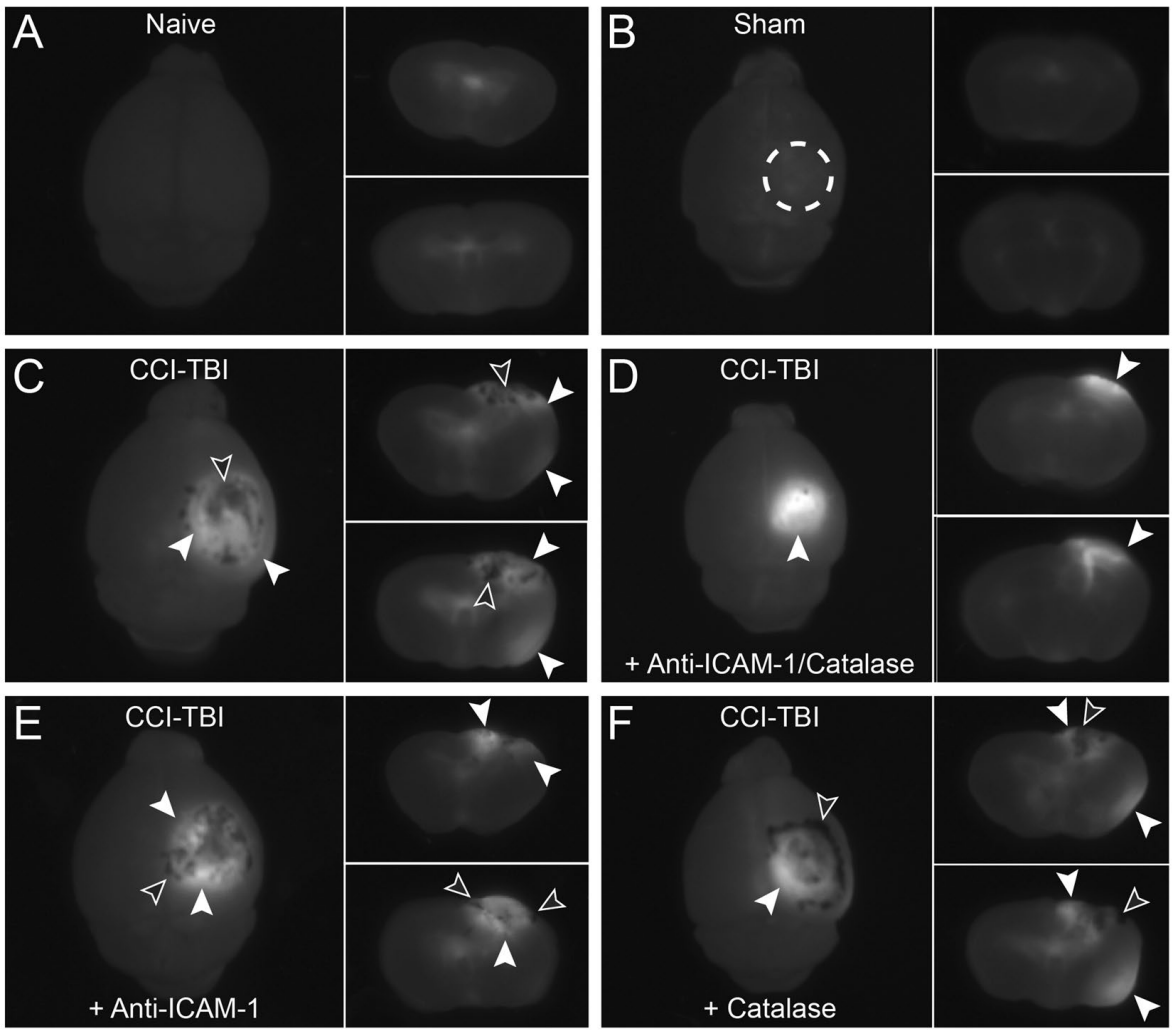
*In vivo* sodium fluorescein assay further elucidates changes in BBB permeability with increased barrier permeability following moderate CCI-TBI and rescue of barrier function with anti-ICAM-1/catalase. Sodium fluorescein was administered IV at 48 hrs post-CCI-TBI to evaluate barrier integrity at this time point. The small molecular weight fluorescent tracer was allowed to circulate for 15 min before tissue perfusion and collection. Fluorescent images captured using short wave UV light demonstrate sodium fluorescein leakage into brain tissue in naive ((**A**), no craniectomy) and sham (**B**) mice and mice sustaining moderate CCI-TBI events at 48 hr following injury (**C**) CCI-TBI only, D: CCI-TBI+Anti-ICAM-1/catalase, (**E**) CCI-TBI+anti-ICAM-1 antibody, and F: CCI-TBI+catalase). The dashed circle in B demarcates where the craniectomy was performed. Sodium fluorescein leakage into the brain extends beyond the area of impact following moderate CCI-TBI, highlighted by white arrowheads. Black arrowheads indicate areas of hemorrhage, edema, or swelling of the brain following injury. Coup and contrecoup mechanisms of injury resulting from CCI-TBI can be appreciated by comparing sodium fluorescein signal detection in the area of impact (top of brain section) to the base of the brain directly opposite of the area of impact in CCI-TBI and CCI-TBI+Catalase groups (**C**,**F**).

Barrier integrity was further investigated using a modified sodium fluorescein (Na-F) permeability assay. At 48 hrs after CCI-TBI, Na-F was introduced by IV injection and allowed to circulate for 15 min prior to perfusion and tissue harvest. Imaged under UV light, the naive condition showed absent Na-F in the brain parenchyma. However, Na-F signal can be detected in the ventricular compartment, as it is able to access the cerebrospinal fluid (Fig. 5A, white dotted line depicts the area of impact when CCI-TBI is delivered). Following CCI-TBI, barrier permeability and vascular disruption permit Na-F escape into the brain parenchyma within and beyond the area of impact (Fig. 5C, white arrows). Contrecoup damage to the brain was observed in CCI-TBI and CCI-TBI+catalase groups (Fig. 5C and F, respectively). TBI is known to result in edema and hematoma formation, events which can be visualized in this assay (black arrowheads). The extent of Na-F leakage into the brain parenchyma was restricted with acute administration of the anti-ICAM-1/catalase conjugate, including the prevention of contrecoup barrier disruption (Fig. 5D), a result not seen with catalase administration alone (Fig. 5E).

### Classical CCI-TBI neuropathology indices are attenuated by anti-ICAM-1/catalase administration

To determine whether neuropathological indices of TBI could be alleviated upon acute administration of anti-ICAM-1/catalase, immunohistochemistry analysis of specific cellular markers was performed at 48 hrs post-CCI-TBI. NeuN was detected to assess neuronal loss following CCI-TBI and anti-ICAM-1/catalase intervention. Glial fibrillary acidic protein (GFAP) was stained to visualize the extent of activation in cortical astrocytes, and ionized calcium binding adaptor molecule 1 (Iba1) was detected to assess changes in microgial morphology and activation state.

**Figure 6:**
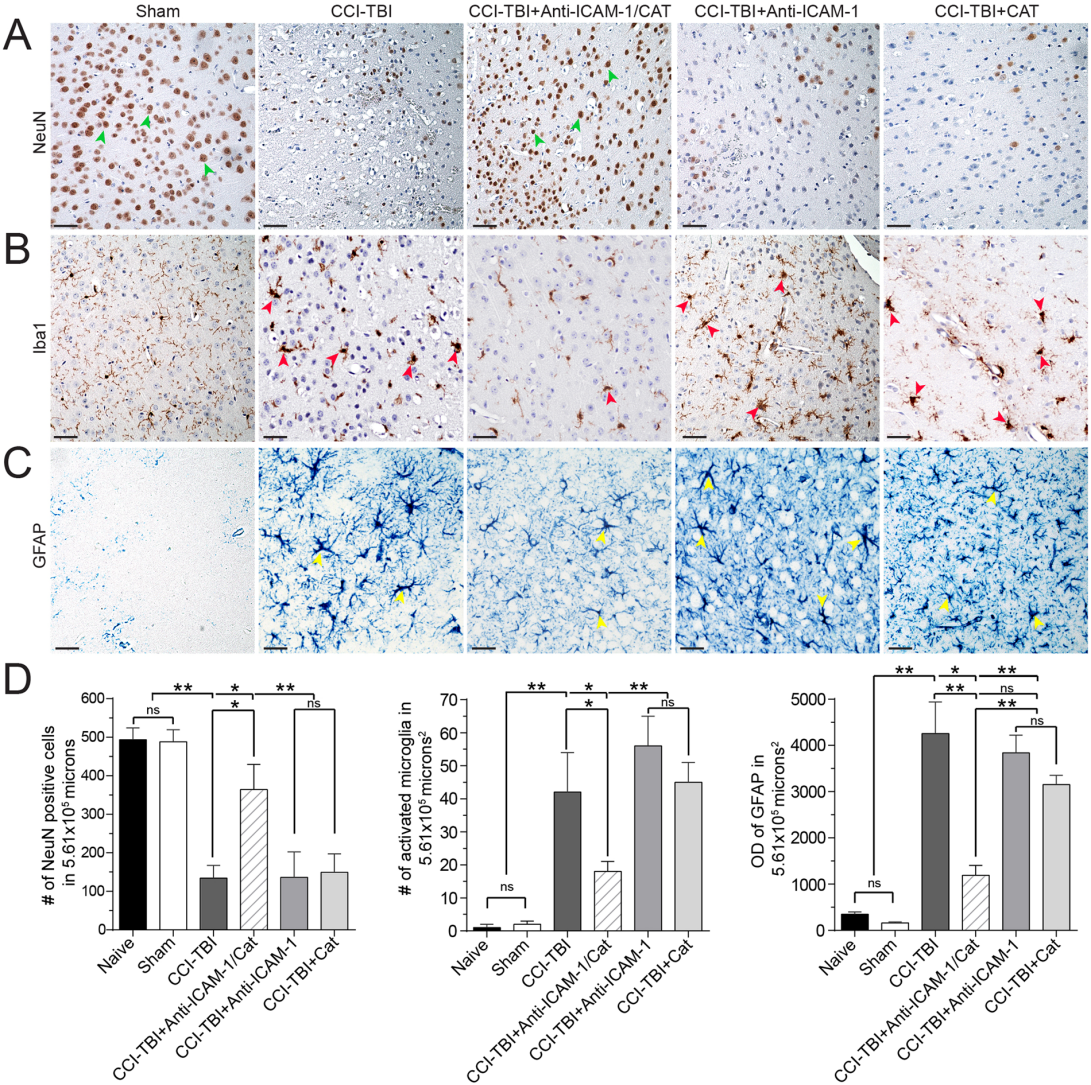
Diminished neuropathology indices with acute anti-ICAM-1/catalase administration following CCI-TBI demonstrated by immunohistochemical staining for neurons, astrocytes, and microglia. (**A**) NeuN staining identifies viable neurons within the cerebral cortex and demonstrates substantial neuronal loss 48 hrs following CCI-TBI. Primary injury mechanisms invariably result in cell death, as indicated by persistently decreased NeuN detection, based on particle count analysis, that is rescued with targeted antioxidant enzyme intervention. NeuN detection following anti-ICAM-1/catalase administration was significantly higher than CCI-TBI alone or in combination with recombinant catalase and anti-ICAM-1. Green arrows point to nuclear stain of NeuN. (**B**) Microglia are finely ramified with small cell bodies in the sham condition. Following CCI-TBI, number of activated microglia per 5.61 × 10^5^ microns squared is significantly increased as indicated by threshold increases in cell body size and Iba1 staining intensity. Anti-ICAM-1/catalase reduced the number of amoeboid microglia per frame, while catalase and anti-ICAM-1 alone did not. Red arrows point to amoeboid microglia. (**C**) GFAP expression indicating astrocyte activation shows significant increase at 48 hrs following CCI-TBI compared to optical density readouts per 5.61 × 10^5^ microns squared for the sham group. Anti-ICAM-1/catalase significantly reduced optical density of GFAP detection at 48 hrs after CCI-TBI compared to CCI-TBI alone. Non-targeted catalase and anti-ICAM-1 controls did not offer benefit regarding astrocyte activation following CCI-TBI, with no significant difference from CCI-TBI alone. Yellow arrows point to astrocytes with robust GFAP staining detection. Scale bars equal 50 microns. (**D**) Associated bar graphs display imaging quantification. Naive animals were included in image analysis and quantification but are not displayed in the montage. Data are presented as mean ± SD. (Ordinary one-way ANOVA. NeuN: F = 39.83, P < 0.0001. Iba1: F = 58.81, P < 0.0001. GFAP: F = 117.8, P < 0.0001).

NeuN staining was used to assess neuronal density and viability 48 hrs after CCI-TBI and treatment with anti-ICAM-1/catalase as a means to determine the degree of neuronal protection from oxidative damage induced cell death pathways (Fig. 6A). NeuN is a neuronal nuclear antigen, commonly used as a biomarker for neurons, and gives distinctive nuclear staining^37^. Cell count per area was measured and compared to naive and sham conditions to reflect neuronal loss with decreased NeuN staining in CCI-TBI. While CCI-TBI resulted in a decreased NeuN-positive cells in the area of impact, including with acute administration of anti-ICAM-1/catalase, neuronal detection was significantly higher with targeted therapy than without, indicating neuronal preservation and reduced neuronal death after CCI-TBI (Fig. 6D).

Iba1 staining allows for the assessment of microglial morphology as these cells undergo drastic phenotypic changes with activation in response to injury^38^. Microglia in the sham condition exhibit small cell bodies and diffusely ramified processes. Following moderate CCI-TBI, Iba1 expression is upregulated and by 48 hrs after injury, microglia undertake an amoeboid-like activated cell shape (Fig. 6B). Number of amoeboid microglia was counted per area of impact and compared between sham, CCI-TBI, CCI-TBI+anti-ICAM-1/catalase, and CCI-TBI+catalase and antibody groups. The number of activated microglia per area was significantly increased following CCI-TBI and reduced toward that of the sham with administration of anti-ICAM-1/catalase but not by catalase or antibody alone (Fig. 6B,D).

GFAP staining was assessed by optical density per area. GFAP expression can be regarded as a sensitive and reliable marker that labels reactive astrocytes responding to injury in the CNS^39^. GFAP staining of the sham condition shows very little baseline expression of GFAP in cortical levels I-V of the mouse brain. Following CCI-TBI, however, GFAP expression level is greatly increased with an approximately 17-fold change in optical density (Fig. 6C,D). GFAP expression level is reduced toward the sham and naive groups with administration of anti-ICAM-1/catalase but not by the catalase or antibody only controls (Fig. 6D). These results suggest a neuroprotective and anti-neuroinflammatory effect of administration of anti-ICAM-1/catalase acutely following CCI-TBI.

### Microglial morphometrics are normalized with administration of anti-ICAM-1/catalase following CCI-TBI

**Figure 7:**
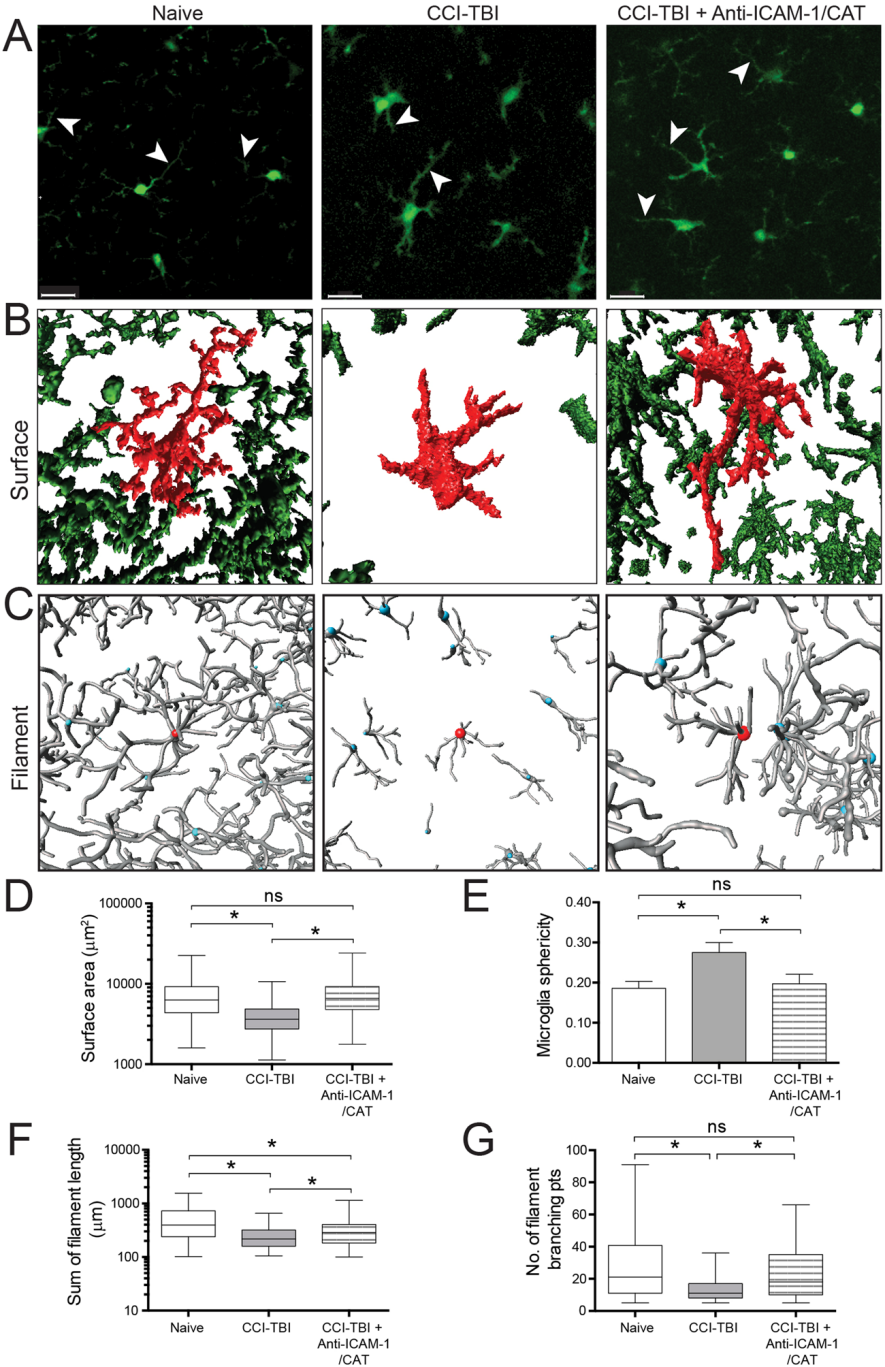
Two-photon imaging detection of microglia in CX3CR1-GFP mice and morphometric analysis of cellular morphology in CCI-TBI and anti-ICAM-1/catalase administration. CX3CR1-GFP mice (B6.129P-*Cx3cr1* ^*tm1Litt*^/J, The Jackson Laboratory) were subjected to moderate CCI-TBI with and without anti-ICAM-1/catalase conjugate administration 30 min after impact. No craniectomy (naive) CX3CR1-GFP mice served as control. At 48 hrs following CCI-TBI, subjects were perfusion fixed, brain tissue was collected and subsequently sectioned into 1 mm segments for two-photon imaging. (**A**) Representative single slice images depict microglial ramification in the area of impact for naive, CCI-TBI, and CCI-TBI+anti-ICAM-1/catalase mice. Arrowheads point to microglial processes, which are finely ramified in the naive condition and thicken and retract following CCI-TBI. Anti-ICAM-1/catalase attenuates changes in microglia cell body enlargement and process retraction. Scale bar 20 μm. Imaris (Bitplane) software was utilized for 3D reconstruction of GFP-expressing microglia from z-stack images obtained by two-photon microscopy. (**B**) Surface rendering was performed for microglia based on GFP signal threshold to measure cellular surface area (**D**) (only objects with surface area >1000 μm^2^ were analyzed) and sphericity (**E**). (**C**) Imaris FilamentTracer was employed to map microglial processes to quantify changes in cell ramification in CCI-TBI. Microglial total filament length (**F**) and number of filament branching points (complexity, (**G**) and were assessed for naive, CCI-TBI, and CCI-TBI+anti-ICAM-1/catalase groups. Data presented at mean ± SD. (Ordinary one-way ANOVA. Surface area: F = 73.12, P < 0.0001. Sphericity: F = 17.20, P = 0.0013. Sum of filament length: F = 74.57, P < 0.0001. Number of filament branching points: F = 38.50, P < 0.0001).

To further delineate inflammatory activity in the cortex following CCI-TBI and possible prevention of inflammatory activation with anti-ICAM-1/catalase administration, microglial morphology was investigated using high power two-photon microscopy to analyze microglial characteristics on the cellular and subcellular scale. Moderate CCI-TBI with or without anti-ICAM-1/catalase treatment was delivered to CX3CR1-GFP mice (The Jackson Laboratory; Bar Harbor, ME), and *ex vivo* brain segments were imaged by two-photon microscopy 48 hrs following injury to evaluate microglia morphology as a correlate of activation state. Single optical slice images obtained during z-stack acquisition revealed structural properties of microglia that can be further studied and rendered by 3D interactive data visualization software (Imaris, Bitplane; Zurich, Switzerland). CCI-TBI results in microglia activation with cell body enlargement and thickening and retraction of processes by 48 hrs following injury. These structural changes are attenuated by acute intervention with anti-ICAM-1/catalase (Fig. 7A). Microglial morphological changes occur grossly at the level of the cell body as well as more striking changes in long ramified processes (Fig. 7A, arrowheads)^40^. Using Imaris image analysis software, microglia were assessed for morphometrics including surface area, sphericity, processes length, and number of process branching points (complexity and ramification). The Imaris surface module was used to determine average microglial surface area and sphericity, a measure of how spherical an object is. Rendering of microglia using the surface module demonstrated enlarged cell bodies and thickened and retracted processes following CCI-TBI and a reversion to the naive phenotype with administration of anti-ICAM-1/catalase (Fig. 7B). With a loss of complexity and ramification that accompanies a change to an amoeboid morphology, mean surface area of microglia significantly decreased, nearly 2-fold, 48 hrs after CCI-TBI compared to naive. Administration of anti-ICAM-1/catalase resulted in no significant difference in surface area at 48 hrs (Fig. 7D). The sphericity of an object, or cell, is defined as the ratio of the surface area of a sphere (with the same volume as the given object) to the surface area of that object^41^. Sphericity was increased by approximately 50% with CCI-TBI, while no significant difference was seen following anti-ICAM-1/catalase (Fig. 7E). The filament module was used to determine the sum of filament or process length per cell and number of process branching points per cell. With approximately the same number of cell bodies per frame, CCI-TBI greatly reduces the extent to which microglial processes survey their environment (Fig. 7C). At 48 hrs following CCI-TBI, mean microglial process length decreased approximately 2-fold (Fig. 7F). This is shifted toward the naive phenotype with anti-ICAM-1/catalase treatment. Number of branching points was decreased with CCI-TBI by greater than 50%. Anti-ICAM-1/catalase prevented this reduction, and microglia showed a mean number of branching points comparable to the naive (Fig. 7G).

## Discussion

The understanding of TBI pathophysiology, particularly the intersecting roles of inflammation and oxidative stress, remains incomplete^42^. TBI results in endothelial activation, BBB damage and permeability^23, 42^, and the production of oxidative stress mediators that contribute to injury propagation in neurotrauma^42, 43^. While studies have shown heterogeneous efficacy of antioxidant therapy, a targeted enzymatic antioxidant approach to TBI management has not yet been explored. We therefore acutely administered endothelial-targeted antioxidant enzyme therapy to study neuropathological outcomes in a mouse model of TBI. Here, we present evidence demonstrating that targeted antioxidant enzyme therapy, conjugates of catalase to anti-ICAM-1 antibodies, conveys protection from TBI-induced BBB permeability, resultant neuropathology, and neuroinflammation.

TBI causes a nearly immediate increase in superoxide production, which initiates the cascade of oxidative stress^19^. Superoxide is quickly dismutated to hydrogen peroxide and oxygen by superoxide dismutase, and hydrogen peroxide, which is more stable than superoxide, is then available to interact with other biological substrates, increasing oxidative damage in the brain^43^. For example, hydrogen peroxide potentiates the release of ferrous iron from hemoglobin extravasated during primary injury vessel disruption and hematoma formation. Catalytically active iron participates in free radical reactions in two ways. First, autoxidation of ferrous iron results in continued formation of superoxide. Secondly, ferrous iron can be oxidized in the presence of hydrogen peroxide to form hydroxyl radicals by the Fenton reaction^43^. Accordingly, the cerebral vasculature has been reported to be the initial source of hydroxyl radical production following TBI^18, 19^. We demonstrate rapid free radical production following CCI-TBI using dihydroethidium in a novel *in vivo* approach to ROS detection. Dihydroethidium is freely permeable and therefore able to detect the production of free radicals in the injured brain. These results coincide with findings that free radical production and superoxide are detrimental to TBI outcome^44^. Further downstream, hydroxyl radical-induced lipid peroxidation is a major mechanism of cellular damage in TBI, and intervening with free radical production at the level of hydrogen peroxide has the potential to eliminate subsequent oxidative reactions and the propagation of injury via this pathway. Therefore, we sought to investigate hydrogen peroxide levels in the injured cortex. To our knowledge, this is the first attempt at a direct readout for hydrogen peroxide in the brain following CCI-TBI. Congruent with previous work, increased levels of hydrogen peroxide were found 4 hrs after CCI-TBI which may reflect increases seen in lipid peroxidation, increased 3-nitrotyrosine levels, and ADP ribosylation as have been demonstrated previously^45–47^. Accordingly, we show increased 3-nitrotyrosine production in our model of CCI-TBI that is reduced upon administration of anti-ICAM-1/catalase. Work from our group and others has suggested that interfering with oxidative stress using the superoxide scavenger, Tempol, attenuates neuroinflammation and BBB permeability^48–50^. Thus, intervention at the level of hydrogen peroxide may too be neuroprotective in TBI.

Another early event following TBI is endothelial activation, with increased expression of cell adhesion molecules, including ICAM-1. ICAM-1 expression following injury is regulated by cytosolic phospholipase A2a via NFkB and CREB transcription factors, which play major roles in the expression of genes that characterize inflammation^51^. We show marked increases in ICAM-1 expression on cortical vasculature up to 48 hrs following CCI-TBI (Fig. 1). Interestingly, we observed increased ICAM-1 expression in the vasculature of subcortical brain regions as well (e.g. hippocampus), comparing ipsilateral to contralateral brain regions in the coronal section shown in Fig. 1. Upregulation of ICAM-1 and other adhesion molecules by the cerebral endothelium following a TBI is well established^52^. Our results support previous findings of early endothelial activation following TBI, including *in vitro* studies^30^ and recent work with the CCI-TBI model^53, 54^. Increased ICAM-1 detection in the hippocampus likely indicates mechanisms of ongoing inflammation in this brain region and may contribute to cognitive decline that occurs in TBI^55^. Therefore, future studies will aim to elucidate the effect of endothelial-targeted antioxidant enzyme therapy on functional outcomes in CCI-TBI.

A hallmark of endothelial activation is an alteration in barrier function. Mechanical injury and the production of inflammatory factors by perivascular cells and brain microvascular endothelial cells affect permeability of the BBB^54^. We assessed BBB integrity following CCI-TBI by fibrinogen staining and a novel *in vivo* permeability assay using Na-F, which is more sensitive to small changes in barrier permeability than the more traditionally used permeability marker, Evans Blue. Following injury, the BBB breaks down and permits the leakage of plasma proteins such as fibrinogen into the brain tissue. Fibrinogen positive staining was detected extensively following CCI-TBI in the cortex and white matter tracks, even extending beyond the area of impact and into the contralateral hemisphere (Fig. 4B), a phenomenon also seen in moderate CCI-TBI by immunostaining for IgG^56^. It has been reported that fibrinogen levels increase following TBI, and that this acute phase protein response can contribute to propagating endothelial inflammation at the BBB^57–59^. To account for the potentially confounding influence of fibrinogen levels, we also studied barrier permeability using Na-F. At only 376.27 Da, Na-F is highly sensitive to BBB hyperpermeability^53, 60, 61^. Interestingly, the Na-F assay was able to detect barrier changes in the area of impact and at the base of the brain, reflecting coup and contrecoup mechanisms of injury. This is known to occur when the force of an impact is sufficient to cause damage between the brain and the skull at the impact site as well as to propel the brain in the reverse direction to collide with the skull on the opposite side of the head^62^. To correlate functional barrier status with BBB structure, we also investigated tight junction protein expression profile in the ipsilateral cortex following CCI-TBI and observed a decrease in tight junction proteins occludin and claudin-5 that was rescued toward the expression levels seen in the sham with acute administration of anti-ICAM-1/catalase. Both fibrinogen and Na-F permeability readouts demonstrated reduced target detection in the injured brain following acute administration of anti-ICAM-1/catalase but not with catalase or anti-ICAM-1 antibody alone. These results suggest that targeting antioxidant enzyme therapy to the activated endothelium conveys BBB protection.

In accord with BBB protection by anti-ICAM-1/catalase, a corresponding reduction in neuropathology indices was anticipated. To assess neuropathology outcomes, neurodegeneration was measured by NeuN staining and glial activation with GFAP and Iba1. Here, NeuN staining revealed a decrease in cortical neuronal detection 48 hrs following CCI-TBI. GFAP was greatly upregulated by cortical astrocytes at 48 hrs following CCI-TBI, with GFAP signal detection present in other brain regions (i.e. hypothalamus), indicating neuroinflammatory changes following CCI-TBI extending beyond the cortex (data not shown). These findings have been presented in our previous work and the work of others, suggesting that TBI can have sustained effects on cognitive function and reward circuitry that may be attenuated by secondary injury intervention^12, 63^. Reactive astrogliosis has been reported up to 60 days following CCI-TBI demonstrating the ongoing inflammatory response of astrocytes to brain injury^64^. While astrocytes are known to contribute to enhanced inflammation in the injured CNS, microglia are the specialized immune cells of the brain^65^. Microglia express high levels of Iba1 and upregulate its expression in activated states such as following TBI^65^. It is generally thought that the appearance of microglia reflects their functional state^66, 67^. Here, we demonstrate an increase in Iba1 detection and number of activated microglia per unit area in the site of injury, as indicated by an enlarged amoeboid-like morphology. Upon activation, microglia retract and thicken their processes, which will decline in number^68^. We studied microglia morphological changes in greater detail using two-photon microscopy. Morphometric analysis revealed that CCI-TBI results in decreased microglial surface area and increased cell sphericity as microglia transition from a resting ramified to an activated amoeboid phenotype. We also noted decreases in total process length and number of process branching points per cell. These findings support the current view of changes in microglial morphology in TBI.

Each of the neuropathological findings discussed are known to occur in animal models of TBI and in humans^34^. Persistent neuroinflammation is a major contributor to the co-morbidities from which many TBI patients suffer, and long-term follow up of patients 10 to 20 years after TBI has provided further evidence of late stage neurodegeneration^69^. Of significance, neuropathological trends for loss of NeuN detection, increased GFAP expression, and changes in microglia morphology were shifted to reflect the sham phenotype with administration of anti-ICAM-1/catalase. While catalase targeted to ICAM-1 conveyed significant barrier and anti-inflammatory neuroprotection to CCI-TBI, non-targeted recombinant catalase and anti-ICAM-1 antibody alone did not. These results are congruent with other studies employing antibody targeted antioxidant enzyme therapy in which targeted strategies provide protective effects unmatched by non-targeted or PEGylated antioxidant enzyme cargo^26–28, 70^. Additionally, a previous study by Knoblach and Faden found functional improvement in rats sustaining TBI followed by repeated treatments with anti-ICAM-1. However, the authors also observed improvement following administration of non-specific IgG control. Treatment with anti-ICAM-1 did reduce cortical myeloperoxidase activity uniquely compared to IgG control^71^. While we did not investigate neutrophil invasion into the brain following TBI, it is possible that anti-ICAM-1/catalase prevents oxidative damage by limiting immune cell infiltration in addition to combating oxidative stress processes at the endothelium.

The results of this study highlight oxidative stress as a point of intervention that may attenuate the damaging pathologies of secondary injury. Future studies will seek to identify the functional significance of antioxidant enzyme-mediated BBB preservation and reduced neuropathology in CCI-TBI. While several antioxidant strategies have been attempted in TBI, the marginal success of such trials has limited the translation of these therapies to clinical practice. Additionally, heterogeneity of antioxidant therapies, treatment regimens, patient populations, and patient TBI severity has prevented clear conclusions and guidelines regarding the use of these therapies in TBI^72^. Emerging platforms in nanomedicine offer widespread implication to the treatment of various disorders; however, further study is warranted regarding their use in TBI^73^. The study of targeted antioxidant enzyme therapy administered acutely following CCI-TBI provides a proof-of-concept perspective of novel treatment paradigms for TBI to combat oxidative secondary injury mechanisms at the blood-brain barrier.

## Methods

### Reagents

Catalase from bovine liver is from Calbiochem (San Diego, CA). Succinimidyl-6-[biotinamido]hexanoate (NHS-LC-biotin), 4-[N-maleimidomethyl]cyclohexane-1-carboxylate (SMCC), and N-succinimidyl-S-acetylthioacetate (SATA) are from Pierce Biotechnology (Rockford, IL). The anti-ICAM-1 monoclonal antibody (mAb) used was mAb YN1/1.7.4, a rat mAb directed against murine Intercellular Adhesion Molecule-1 (ICAM-1)^74^. Amino-chemistry methods for preparing anti-ICAM-1/catalase conjugates were adapted for the antibody (anti-ICAM-1) and enzyme (catalase) employed for this study as detailed previously^75, 76^.

### Vertebrate animals and controlled cortical impact traumatic brain injury model

The Institutional Animal Care and Use Committee (IACUC) at Temple University (Philadelphia, PA) approved all procedures detailed in this study that required the use of vertebrate animals prior to initiating any experimental objectives. Additionally, all methods were performed in full compliance with Temple University’s IACUC policies and the National Institutes of Health (NIH) ethical guidelines. Animals were housed and allowed to acclimate for 1–2 weeks in the Temple University Central Animal Facility. The animals were provided standard environmental enrichment conditions and were fed with a commercial pellet diet and water *ad libitum*. Eight-week-old male C57BL/6 J and CX3CR1-GFP mice (B6.129P-Cx3cr1tm1Litt/J) were obtained from The Jackson Laboratory (Bar Harbor, ME). Animals were anesthetized using 5% (induction) and 2% (maintenance) isoflurane in oxygen, and the controlled cortical impact surgical procedure was followed as previously described^30^. Briefly, a 4 mm craniectomy was performed over the right somatosensory cortex between bregma and lambda suture lines. A moderate TBI (CCI-TBI) was delivered at 3.5 m/s to a depth of 1 mm using an Impact One™ Stereotaxic CCI Instrument (Leica Microsystems; Buffalo Grove, IL) outfitted with a 2 mm diameter piston. The dwell time was 0.5 s. After impact, the craniectomy was sealed with a 5 mm glass coverslip (Electron Microscopy Sciences; Hatfield, PA) to allow for monitoring of the impact site^12^. All animals were individually housed following the CCI-TBI procedure. Naive animals did not undergo any surgical intervention. Conjugates (100 μg anti-ICAM-1/catalase), non-conjugated antioxidant enzymes (100 μg recombinant catalase), or anti-ICAM-1 antibody (62 μg monoclonal YN1 - molar equivalent to conjugate) were administered by retro-orbital injection at 30 min following CCI-TBI. Experimental groups were as follows: non-craniectomized controls (naive), surgical shams (craniectomy without TBI), CCI-TBI only, CCI-TBI+catalase, CCI-TBI+anti-ICAM-1 antibody, CCI-TBI+anti-ICAM-1/catalase.

### Immunohistochemistry

Immunohistochemistry was performed on brain tissue segments to evaluate the extent of neuroinflammation and neuropathology in all experimental groups. At appropriate time points, anesthetized mice were transcardially perfused with PBS followed by Poly/LEM fixative (Polysciences, Inc.; Warrington, PA). Perfused brains were removed from the skull and placed in Poly/LEM fixative for 24 hrs at 4 °C. Brains were dissected into 2 mm segments using a stainless steel brain matrix (CellPoint Scientific, Inc.; Gaithersburg, MD). Segments were post-fixed in Poly/LEM fixative at 4 °C for an additional 24 hrs. Next, segments were washed with PBS, processed using a Tissue-Tek® VIP® 6 (Sakura Finetek USA, Inc.; Torrance, CA), paraffin-embedded using a TN-1500 Embedding Console System (Tanner Scientific, Inc.; Sarasota, FL), and sectioned using a rotary microtome (Leica Microsystems, Inc.; Buffalo Grove, IL). 5 μm paraffin-embedded sections from each experimental group (naive, sham, CCI-TBI only, CCI-TBI+anti-ICAM-1/catalase, CCI-TBI+catalase, CCI-TBI+anti-ICAM-1 antibody) were cleared, rehydrated and stained for NeuN, GFAP, Iba1, Fibrinogen, and 3-nitrotyrosine to determine neuronal survival, degree of astrogliosis, microglial activation, degree of vascular permeability, and oxidative stress respectively, in the region of impact. A time course of post-CCI-TBI only samples was stained for ICAM-1 to determine its expression profile in cerebral vasculature. Prior to primary antibody incubation, sections stained for GFAP, Iba1 and ICAM-1 were HIER pre-treated with 10 mM citric acid buffer (pH 6.0), while those stained for Fibrinogen were PIER pre-treated with Proteinase K (Dako). Sections stained for NeuN received no pre-treatment. All sections were incubated in primary antibody prepared in Dako Antibody Diluent either for 1 hr at RT (NeuN, Iba1, Fibrinogen, 3-NT), or O/N at 4 °C (GFAP and ICAM-1) at the following dilutions: NeuN (1:500, Covance), Iba1 (1:400, Wako Chemicals), Fibrinogen (1:400, Dako), GFAP (1:2000, Cell Signaling), 3-NT (1:1000, Abcam) and ICAM-1 (1:250, Sino Biologicals). Positive antibody staining was detected using an HRP- or AP-conjugated labeled polymer system (ImmPRESS Staining Kits, Vector Laboratories) and subsequently visualized using Sigma DAB (NeuN and Iba1), Vector DAB (Fibrinogen), Dako DAB+ (ICAM-1 and 3-NT) or Vector Blue (GFAP). Sections were then dehydrated and cover slipped in preparation for imaging.

### *In vivo* free radical detection by dihydroethidium staining

Dihydroethidium (Thermo Fisher Scientific, Inc.; Waltham, MA) was administered by intraperitoneal (IP) injection in at 6 μg/g body weight to mice 3 hrs following CCI-TBI. The probe circulated for 1 hr before PBS perfusion and brain harvest. Tissue was fresh frozen in optimal cutting temperature compound. 20 μm frozen sections were imaged for red fluorescence by epifluorescence microscopy and analyzed by pixel intensity profile using NIS Elements imaging software (Nikon, Japan).

### Hydrogen peroxide detection assay

Using the commercially available OxiSelect™ Hydrogen Peroxide/Peroxidase Assay Kit (Cell Biolabs, Inc.; San Diego, CA), hydrogen peroxide levels were measured in mouse cortical tissue collected 4 hrs following CCI-TBI or from naive and sham controls. Mice were euthanized by isoflurane overdose and decapitated to quickly remove the brain. On ice, the ipsilateral cortex was isolated and homogenized using a 2 ml Dounce manual homogenizer (Sigma-Aldrich; St. Louis, MO) in 200 μl tissue lysis buffer CelLytic™ MT Cell Lysis Reagent (Sigma-Aldrich; St. Louis, MO). Homogenate was centrifuged at 10,000 × g for 5 min at 4 °C, and supernatant was transferred to a new tube on ice. Sample aliquots were combined with the provided ADHP/HRP probe, incubated at room temperature for 30 min and read on a Spectramax M5 fluorescence plate reader (Molecular Devices; Sunnyvale, CA) (excitation 535 nm, emission 595 nm, auto cut-off 590 nm). Samples were read in duplicate and data are presented as relative fluorescent units (RFU) mean ± S.D. with n = 4 samples per condition.

### Western blot analysis

At 48 hrs following CCI-TBI, n = 3 mice were perfused with 20 ml PBS and brains harvested for sham, CCI-TBI, and CCI-TBI+anti-ICAM-1/catalase groups. The ipsilateral cortex was isolated and immediately homogenized in 400 μl lysis buffer (CelLytic MT, Sigma) including protease and phosphatase inhibitors. Samples were loaded onto a 4–20% Mini-Protean TGX gel (Bio-Rad, Hercules, CA). Gels were transferred to nitrocellulose membranes using the Trans-blot Turbo™ transfer system (Bio-Rad) following the manufacturer’s protocol. Following transfer, membranes were stained with Ponceau S (Sigma) to visualize total protein. According to Precision Plus Protein Standards (Bio-Rad) loaded in lane 1, membranes were cut at 75 kDa and between 25 and 37 kDa to conserve antibody use. Membranes were briefly washed with diH_2_O. Membranes were blocked with SuperBlock (Bio-Rad) and all primary and secondary antibodies were resuspended in SuperBlock. Primary and secondary antibodies were resuspended in SuperBlock and used as follows: occludin (abcam, Cambridge, UK, rabbit, 1:1000)^77^, Claudin-5 (Novus Biologicals, Littleton, CO, rabbit, 1:1000)^78^, GAPDH (abcam, rabbit, 1:1000)^79^, rabbit-HRP (GE Healthcare, Princeton, NJ, 1:10,000). HRP-conjugated antibodies were detected using Supersignal West Pico chemiluminescent substrate (Thermo Fisher Scientific) and visualization of luminescent signal was obtained using the gel documentation system, G:Box Chemi HR16 (Syngene, Frederick, MD). Densitometry was performed with the image analysis software, Image J 1.48 v (NIH).

### *In vivo* sodium fluorescein blood-brain barrier permeability assay

Changes in BBB permeability were assessed using the small molecular weight fluorescent tracer, sodium fluorescein (Na-F, Sigma-Aldrich; St. Louis, MO); the procedure performed was a modification of previously described methods^53^. Briefly, animals were injected retro-orbitally with 100 μl of 5% Na-F in PBS. The tracer circulated for 15 min. The mice were anesthetized and transcardially perfused with 20 ml PBS. The animals were then decapitated and the brains quickly removed from the skull and placed into ice cold PBS. Meninges were removed, and the brain was visualized whole from the superior surface under short-wave UV light using a G:Box gel and blot imaging system (Syngene; Frederick, MD). Brains were then segmented through the impact site and imaged again to better visualize the underlying extent of BBB disruption.

### Two-photon microscopy of CX3CR1-GFP microglia and morphometric analysis

Eight-week-old male CX3CR1-GFP mice (B6.129P-Cx3cr1tm1Litt/J) were obtained from The Jackson Laboratory and randomly designated as non-craniectomized controls (naive) or for moderate CCI-TBI with or without acute conjugate administration (n = 3 per group). At 48 hrs post-injury, mice were transcardially perfused with PBS followed by Poly/LEM fixative. Brains were post-fixed whole in Poly/LEM fixative for 48 hrs at 4 °C and the impact site was dissected into 1 mm segments for imaging. Two-photon microscopy was performed using a TCS SP5 II MP microscope (Leica Microsystems, Inc.) configured with a tunable femto-second pulsed Mai Tai Ti-Sapphire laser (Spectra-Physics; Santa Clara, CA). A 20X water immersion objective (HCX APO L NA 0.95) was used to visualize each specimen, and z-stacks were acquired using LAS imaging software (Leica Microsystems, Inc.). Images were acquired using an 890 nm excitation wavelength with 0.5 μm resolution. Intrinsic GFP signal was detected by a non-descanned detector using a FITC-TRITC filter set consisting of a dichroic beamsplitter (BS 560) and two bandpass filters (BP 525/50 and BP 585/40). Z-stacks were obtained at 200 Hz and 1024 × 1024 pixels per image frame without compensation to a depth of 250 μm. Z-stacks were analyzed using Imaris 8.1.2 (Bitplane; Zurich, Switzerland). The Imaris surface module was used to determine average microglial surface area and sphericity per region of interest (ROI) per z-stack. The filament module was adapted for application to microglia to determine sum of filament length and number of filament branching points per object. Objects were identified with surface area detail of 0.1 μm and absolute threshold intensity 25.0. Results were filtered to exclude objects with surface area below 1000 μm^2^, as these values can represent cell fragments identified by the software as objects. Results are presented as mean surface area (μm^2^), mean sphericity, or mean sum of filament length (μm) ± S.D.

### Bright-field and epifluorescence microscopy and image analysis

Fluorescent images were acquired using a Coolsnap EZ CCD camera (Photometrics; Tucson, AZ) connected to an Eclipse 80i microscope (Nikon Instruments, Inc.; Melville, NY) with a solid-state Lumencore SOLA light engine®. Equal capture parameters were used to acquire fluorescent images from all samples. ImageJ software (1.48 v http://rsb.info.nih.gov/ij/; Bethesda, MD) was used to threshold for fluorescence intensity and to convert to binary images. For analysis of chromogen immunostaining of ICAM-1, GFAP, Iba1, NeuN, and 3-NT, ImageJ analysis was performed by batch processing with an automated cell-counting macro. The macro was configured to perform particle counting on high resolution images (5/sample, 3/group, taken at 20X objective magnification) on immunolabeled cells. The images were calibrated and then sequentially processed for background subtraction, color threshold segmentation, and binary conversion (with application of the watershed function). Cells identified from the above parameters in an area of 5.61 × 10^5^ μm^2^ were counted by use of the analysis particles function (selected by particle area and circularity).

### Statistical analysis

Data were analyzed for statistical significance using Prism software (version 6 GraphPad Software Inc.; La Jolla, CA). Student’s t-tests and one-way analysis of variance (ANOVA) with Dunnett’s post-hoc tests were performed to analyze the biochemical assays and ICAM-1 time course staining. IHC was analyzed by one-way ANOVA followed by Tukey’s post-hoc tests. Two-photon imaging was analyzed by one-way ANOVA with Tukey’s post-hoc tests. For all tests, statistical significance was defined at p < 0.05.

## Electronic supplementary material

Supplemental Figure 1
