# Supplementary material for: Acute administration of catalase targeted to ICAM-1 attenuates neuropathology in experimental traumatic brain injury
Source: Sci Rep. 2017 Jun 19;7:3846. doi: 10.1038/s41598-017-03309-4 (PMC5476649; doi:10.1038/s41598-017-03309-4)
Supplement: Supplementary file 1 — Supplemental Figure 1 [file 41598_2017_3309_MOESM1_ESM.pdf]

Supplemental Information

Acute administration of catalase targeted to ICAM-1 attenuates neuropathology in experimental traumatic brain injury

Authors: Evan M. Lutton, Roshanak Razmpour, Allison M. Andrews, Lee Anne Cannella, Young-Jin Son, Vladimir V. Shuvaev, Vladimir R. Muzykantov, and Servio H. Ramirez

The following gages include:

Supplemental Figure 1

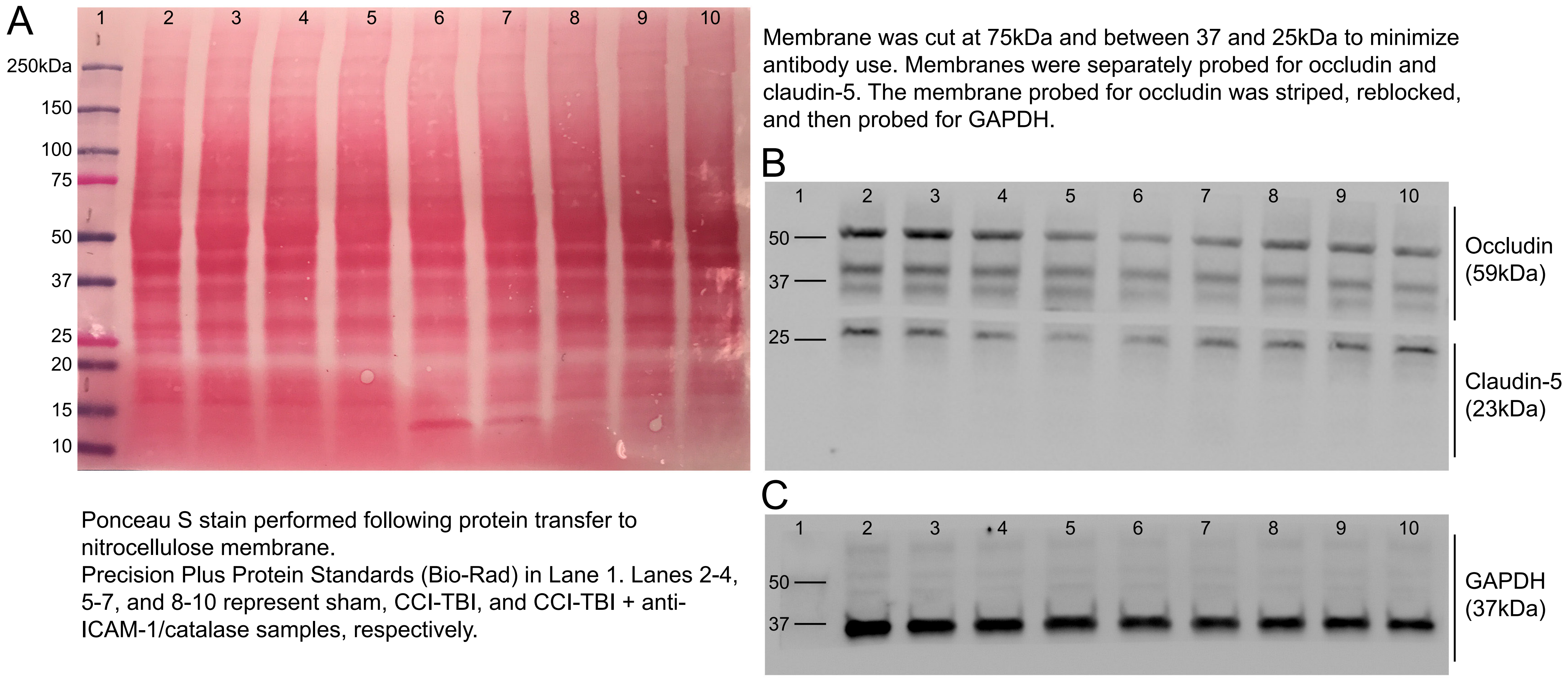

Supplemental Figure 1. Display of full length blots for occludin, claudin-5, and GAPDH Western blot analysis. A) Ponceau S staining of full length blot following protein transfer to nitrocellulose membrane. Precision Plus Protein Standards (Bio-Rad) in Lane 1. Lanes 2-4, 5-7, and 8-10 represent sham, CCI-TBI, and CCI-TBI + anti-ICAM-1/catalase samples, respectively. B) Membrane was cut at 75kDa and between 37 and 25kDa to minimize antibody use. Membranes were separately probed for occludin and claudin-5. C) The membrane probed for occludin was striped, reblocked, and then probed for GAPDH.
